# The Activin/FLRG Pathway Associates with Poor COVID-19 Outcomes in Hospitalized Patients

**DOI:** 10.1128/MCB.00467-21

**Published:** 2022-01-20

**Authors:** Megan McAleavy, Qian Zhang, Peter J. Ehmann, Jianing Xu, Matthew F. Wipperman, Dharani Ajithdoss, Li Pan, Matthew Wakai, Raphael Simonson, Abhilash Gadi, Adelekan Oyejide, Sara C. Hamon, Anita Boyapati, Lori G. Morton, Tea Shavlakadze, Christos A. Kyratsous, David J. Glass

**Affiliations:** a Regeneron Pharmaceuticals Inc., Tarrytown, New York, USA

**Keywords:** activin A, activin B, FLRG, FSTL3, SARS-Cov-2, COVID-19, acute respiratory disease syndrome, ARDS

## Abstract

A subset of hospitalized COVID-19 patients, particularly the aged and those with comorbidities, develop the most severe form of the disease, characterized by acute respiratory disease syndrome (ARDS), coincident with experiencing a “cytokine storm.” Here, we demonstrate that cytokines which activate the NF-κB pathway can induce activin A. Patients with elevated activin A, activin B, and FLRG at hospital admission were associated with the most severe outcomes of COVID-19, including the requirement for mechanical ventilation, and all-cause mortality. A prior study showed that activin A could decrease viral load, which indicated there might be a risk to giving COVID-19 patients an inhibitor of activin. To evaluate this, the role for activin A was examined in a hamster model of SARS-CoV-2 infection, via blockade of activin A signaling. The hamster model demonstrated that use of an anti-activin A antibody did not worsen the disease and there was no evidence for increase in lung viral load and pathology. The study indicates blockade of activin signaling may be beneficial in treating COVID-19 patients experiencing ARDS.

## INTRODUCTION

In the setting of infection by the SARS-CoV-2 virus, it was reported quite early that hospitalized and ICU patients were producing a “cytokine storm” (1), including the cytokines interleukin-1α (IL-1α) and tumor necrosis factor alpha (TNF-α). Clinical studies have demonstrated that blockade of cytokine signaling and steroid treatment are beneficial in improving outcomes in patients; however, further elucidation of downstream signaling pathways contributing to clinical sequelae is important to benefit patients suffering the worst symptoms of COVID-19.

We had previously studied IL-1α and TNF-α in the setting of skeletal muscle cachexia, where these cytokines have been shown to induce skeletal muscle atrophy (2, 3). In one of our prior studies, we determined that IL-1 and TNF-α could induce the production of activin A in skeletal muscle, and that the activin A itself induced skeletal muscle atrophy. We felt this was relevant to COVID-19, because it had been reported separately, back in 2012, that patients who had acute respiratory disease syndrome (ARDS), had high levels of activin A in their bronchial alveolar lavage fluid (4), and, in a preclinical model, this same group found activin A to be sufficient to induce a phenotype reminiscent of ARDS when overexpressed in the trachea via an adenovirus (4). A separate group followed up in 2019, on a distinct ARDS population, and were able to show that activin A and its downstream pathway marker, FLRG, were upregulated in human serum (5).

In addition, the most severe symptoms associated with COVID-19 seem to be age-related; older patients and those with particular comorbidities, like COPD, are more likely to experience ARDS and are at higher risk for mortality from the virus (6, 7). It is therefore of interest to determine molecular mechanisms which are themselves age-perturbed, including the activin A pathway, which might help to explain this correlation of aging with COVID-19-induced mortality.

For these reasons, we studied sera from COVID-19 hospitalized patients to determine if they too had elevated levels of activin A, evidence of activin A pathway elevation, and correlation to activin B and FLRG levels. In addition, another marker previously associated with ARDS, PAI-1, was also evaluated as it is one of the parameters confirmed in the ARMA and ALVEOLI trials associated with ARDS mortality (8, 9). We further sought to determine if the levels of activin A, its pathway marker FLRG as it is activin A activation of Smad2/3 (10) activin B, FLRG, and PAI-1 correlated with important disease markers of COVID-19, such as disease severity, the requirement for supplemental oxygen, other signs of ARDS, and mortality. On a mechanistic level, we were then interested to see if cell types relevant to ARDS and COVID-19, including bronchial and pulmonary smooth muscle, similarly responded to inflammatory cytokines induced by the cytokine storm, to produce activin A, and, if so, by which signaling pathway.

We had performed a clinical trial on COVID-19 patients using a Regeneron anti-IL-6R antibody (sarilumab) (https://clinicaltrials.gov/ct2/show/NCT04315298). We evaluated sera from these patients after randomization and prior to therapy, to determine baseline activin A, activin B, FLRG, and PAI-1 levels, and correlated these to baseline clinical and laboratory variables and important disease outcomes. While we were preparing the manuscript, another manuscript appeared demonstrating activin A and activin B are elevated in COVID-19 patients (11). Our paper is consistent with findings in that manuscript, and goes further in demonstrating mechanism, additional clinical correlations, and providing a preclinical intervention study that helps to derisk this potential treatment approach.

## RESULTS

### Activin A, FLRG, and PAI-1 are elevated in critical patients relative to severe patients or healthy controls.

COVID-19 presents a full spectrum of disease severity, from asymptomatic to mild cold-like symptoms to more disabling but ambulatory illness to more severe illness requiring degrees of hospitalization and intensive care unit (ICU) care, including increasing levels of oxygen support or ventilation. To evaluate the relationship between activin pathway engagement and stages of severe disease progression, we examined the levels of activin A and B and their pathway marker FLRG in sera from COVID-19 hospitalized pneumonia patients with varying disease severity. Baseline samples were collected from a randomized phase 2/3 study of individuals hospitalized requiring low to high supplemental oxygen who were receiving standard of care and supportive therapies. Patients were randomized to either placebo or sarilumab treatment. Serum samples were analyzed in patients following randomization and prior to treatment. To contextualize levels in COVID-19 patients, age-matched sera from healthy controls were also evaluated.

In a prior study, it was shown that activin A can be found in the bronchial alveolar lavage fluid and in the sera of other, non-COVID-19, ARDS patients (4). In a preclinical model, it was further shown that, when overexpressed, activin A was sufficient to phenocopy ARDS (4). Therefore, we sought to determine if activin A and its pathway marker FLRG, are elevated in severe and ICU COVID-19 patients in comparison to healthy controls. Additionally, PAI-1 was studied because it is involved in the coagulation pathway and has been implicated in ARDS (12), so it seemed reasonable to use it as an additional queried biomarker of interest in COVID-19 due to its multiple roles in inflammation, coagulation, as well as an activin-response protein, to determine if there was anything unique about the activin/FLRG pathway.

### Baseline demographics of biomarker study population.

Demographic and health characteristics of the COVID-19 patients studied are shown in Table 1. From the overall clinical trial, which enrolled 1946 patients from 62 sites, the present analysis includes a random subset of 313 COVID-19 patients from 49 sites in addition to 153 age-matched control subjects. Patients in the critical disease stratum had higher percentage of patients admitted to the ICU and receiving steroids or vasopressors compared to patients in the severe stratum (Table 1).

**TABLE 1:** Demographic information and medical history grouped by disease severity

	Result^*a*^ for COVID group			
Parameter	Control (*n* = 153)	Severe (*n* = 62)	Critical (*n* = 251)	*P* ^*b*^
Demographic				
No. female	94 (61.4)	17 (27.4)	76 (30.2)	0.77
No. male	59 (38.6)	45 (72.6)	175 (69.8)	0.77
Age (yrs)	60.0 (53.0–70.0)	55.5 (43.3–70.0)	59.0 (49.5–68.0)	0.06
BMI (kg/m^2^)		28.3 (25.9–34.4)	30.4 (27.3–35.0)	0.15
Hispanic or Latino		11 (17.7)	79 (31.5)	0.04
Race
Asian		7 (11.3)	13 (5.2)	0.14
Black		8 (12.9)	37 (14.8)	0.87
White		28 (45.2)	112 (44.6)	0.99
Other		11 (17.7)	25 (10.0)	0.13
Not reported		8 (12.9)	64 (25.5)	0.05
Medical history				
Days between diagnosis and enrollment		2 (1–5)	4 (2–6)	0.02
Duration of pneumonia prior to enrollment (days)		9 (6–12)	9 (5–13)	0.35
Admitted to ICU prior to enrollment		8 (12.9)	169 (67.3)	<0.0001
Fever		36 (58.1)	122 (48.6)	0.23
Immunocompromised		3 (4.8)	4 (1.6)	0.25
Obesity		24 (38.7)	128 (51.0)	0.10
Hypertension		24 (38.7)	120 (47.8)	0.25
Diabetes		5 (8.1)	47 (18.7)	0.08
Steroid use		4 (6.5)	71 (28.3)	0.0006
Immunosuppressant use		2 (3.2)	7 (2.8)	0.99
Vasopressor use		2 (3.2)	38 (15.1)	0.02

a Summary statistics are presented as median (IQR) for continuous variables and count (percent) for categorical variables.

b Significance computed between severe and critical COVID patients only.

### Activin A, activin B, FLRG, PAI-1 not affected by sarilumab treatment relative to placebo.

Although the clinical data analysis focused on pretreatment sera analysis, we tested for potential pharmacodynamic effects of sarilumab on activin A, activin B, FLRG, and PAI-1 that may confound analysis of longitudinal outcomes. A subset of patients (*n* = 182) enrolled in the phase 3 critical disease strata (*n* = 95 placebo, *n* = 87 sarilumab [400 mg]) were analyzed at baseline along with samples at study day 4 (*n* = 176; *n* = 92 placebo, *n* = 84 sarilumab [400 mg]) and/or study day 7 (*n* = 143; *n* = 59 placebo, *n* = 84 sarilumab [400 mg]). For all four analytes, there was no significant difference between treatment arms at baseline and change from baseline at study days 4 and 7 (data not shown).

### Activin A, activin B, and FLRG are elevated in patients with greater disease severity.

**FIG 1:**
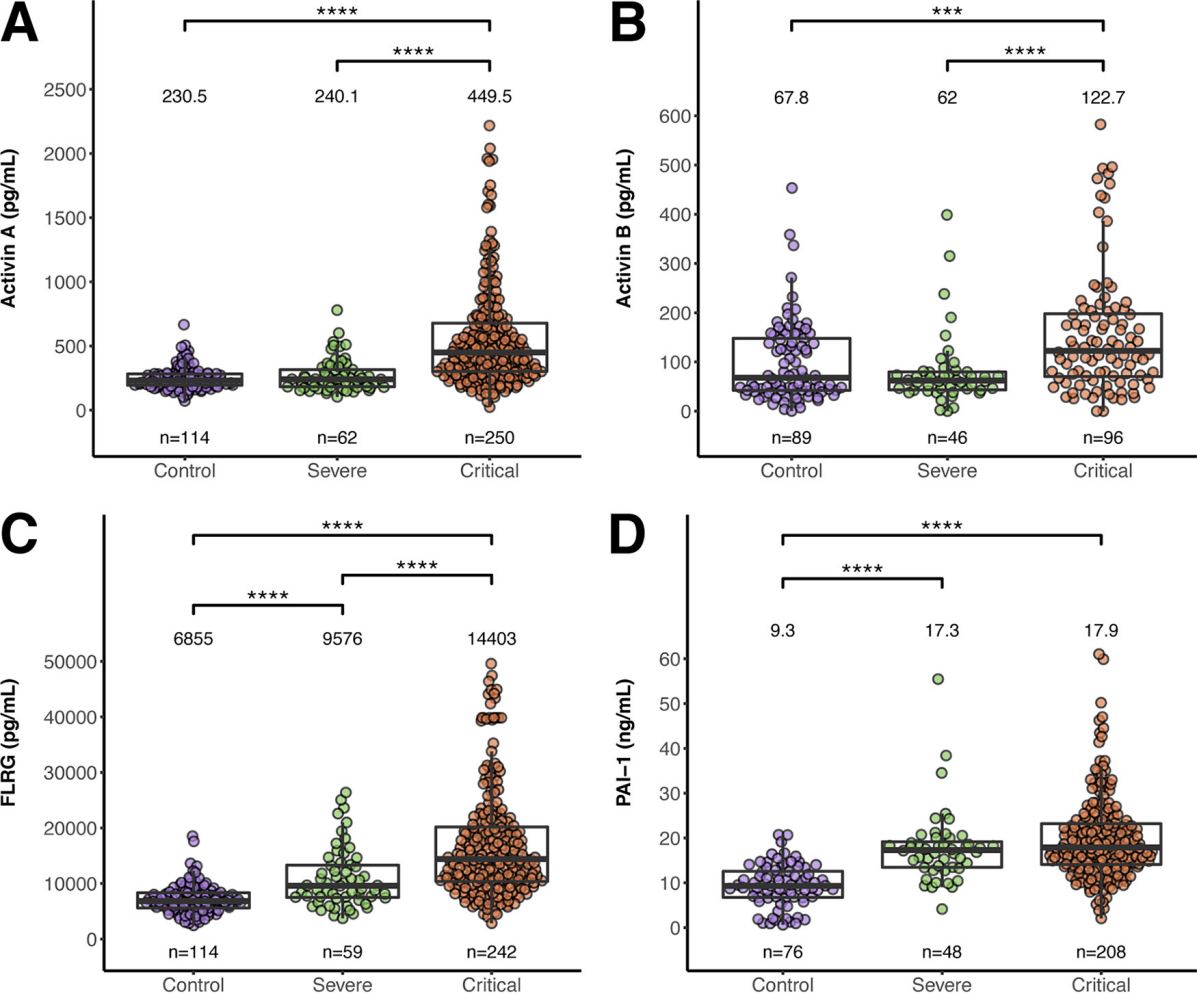
Activin A, activin B, FLRG, and PAI-1 levels versus disease severity in COVID-19 patients prior to dosing and in non-COVID-19 controls. Activin A (A), activin B (B), FLRG (C), and PAI-1 (D) for control subjects, severe COVID-19 patients, and critical COVID-19 patients. Number of patients tested in each group (n) is indicated under each respective plot. Median values for each group are shown above each respective plot. Significant follow-up Dunn pairwise comparisons are shown above each plot; ***, *P* < 0.001, ****, *P* < 0.0001.

Differences were observed between three categories of disease severity (control subjects, severe COVID-19 patients, critical COVID-19 patients) for activin A, activin B, FLRG, and PAI-1 (Fig. 1) (*P* < 0.0001). Follow-up pairwise testing revealed significantly elevated activin A in critical (ICU) COVID-19 patients, compared to severe non-ICU COVID-19 patients and control subjects; severe non-ICU COVID-19 patients and control subjects did not differ in activin A, indicating that activin A is a biomarker for the critical, ICU patients. Similar results were observed for activin B. FLRG levels were also significantly elevated with increased severity of disease; control, severe, and critical indicating that this activin pathway marker can also distinguish between the distinct disease categories. In contrast, for PAI-1, levels were not statistically different between the two COVID-19 strata: severe and critical. However, both were significantly elevated compared to control subjects. These data demonstrate that activin A and B and their pathway marker FLRG are upregulated in more severe settings of COVID-19, where patients require more supplemental oxygen and treatment in the ICU. In contrast, PAI-1 was elevated in both COVID-19 settings and did not distinguish between the severe and critical state.

### Higher levels of activin A, activin B, and FLRG are associated with increased risk of death and greater oxygen requirements at baseline.

**FIG 2:**
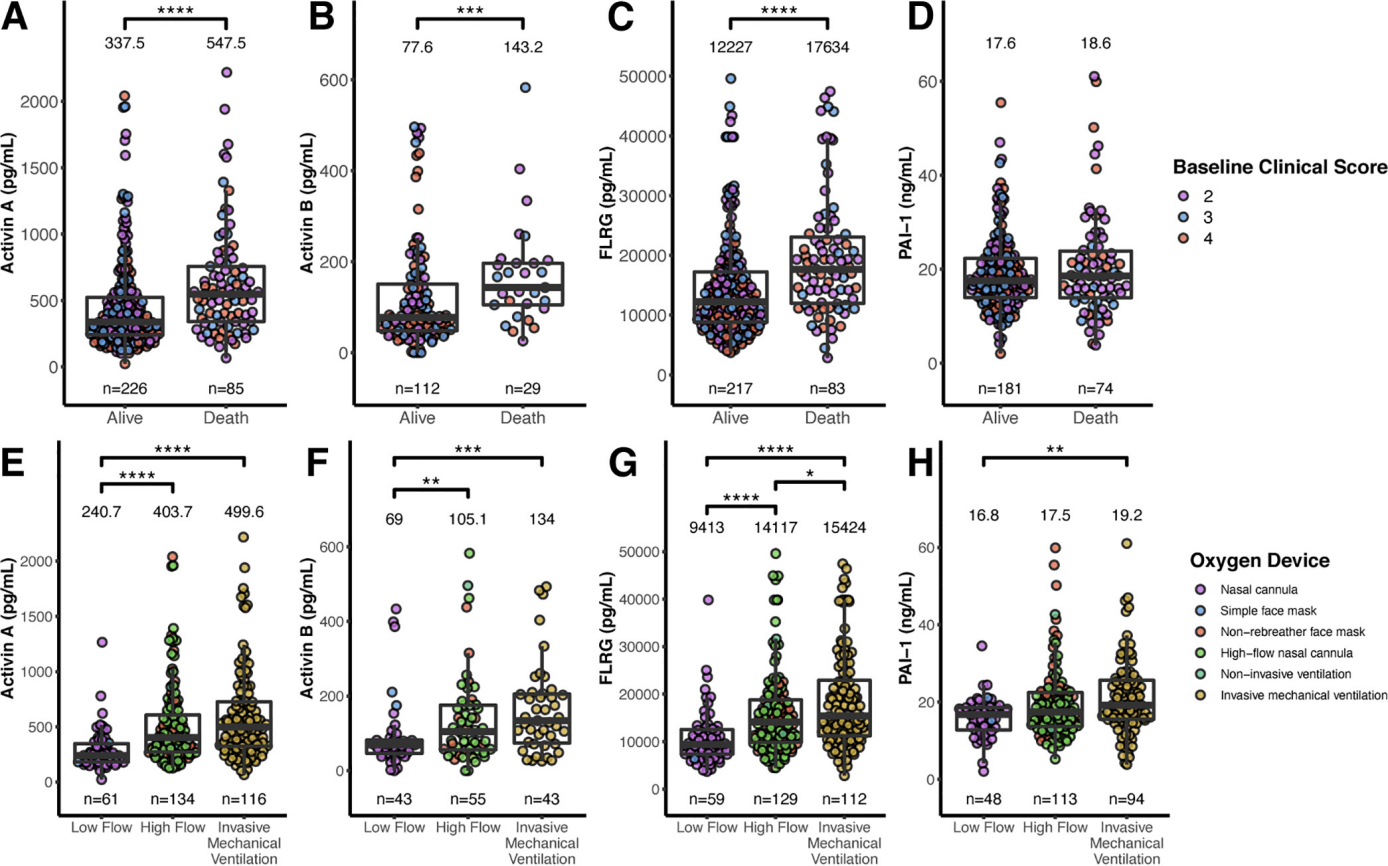
Activin A, activin B, and FLRG levels associate with mortality outcomes and supplemental oxygenation requirements at baseline. (A to D) Activin A (A), activin B (B), FLRG (C), and PAI-1 (D) at baseline between COVID-19 patients who survived and died during the study. (E to H) Activin A (E), activin B (F), FLRG (G), and PAI-1 (H) at baseline between COVID-19 patients requiring various levels of supplemental oxygen. Number of patients tested in each group (n) is indicated under each respective plot. Median values for each group are shown above each respective plot. Significant follow-up Dunn pairwise comparisons are shown above each plot; *, *P* < 0.05, **, *P* < 0.01, ***, *P* < 0.001, ****, *P* < 0.0001.

Since activin/FLRG levels were most highly elevated in critical COVID-19 patients we were further interested in examining the relationship between all-cause mortality and baseline activin A, activin B, FLRG, and PAI-1 in COVID-19 patients (Fig. 2A to D). Baseline activin A was elevated in patients who died compared to patients who survived and was a significant predictor of mortality (OR = 1.54, 95% confidence interval [CI] = 1.21 to 1.96, *P* = 0.0005). The same trend was observed for the downstream marker FLRG (OR = 1.68, 95% CI = 1.31 to 2.16, *P* < 0.0001), consistent with activin A and FLRG as being part of the same pathway. In contrast, baseline PAI-1 was not predictive of mortality (OR = 1.19, 95% CI = 0.91 to 1.54, *P* = 0.20). Activin B was elevated in patients who died but was not a significant predictor of mortality (OR = 1.43, 95% CI = 0.99 to 2.07, *P* = 0.06).

Activin pathway biomarkers were also evaluated in patients requiring various amounts of supplemental oxygen at study start. Oxygen requirements at baseline were stratified into three categories based on oxygen device type: low flow, high flow, and invasive mechanical ventilation (IMV). Data are plotted in Fig. 2E to H. These data indicate that activin A/B and its pathway marker, FLRG, correlate with need for greater supplemental oxygen requirements, but PAI-1, which is elevated in COVID-19 patients relative to healthy subjects, is not linked with the need for greater oxygen in hospitalized patients.

In addition to activin A/B and FLRG, disease progression and clinical outcomes also were related to supplemental oxygen requirements upon study enrollment (Table 2). Patients requiring greater supplemental oxygen pretreatment experienced more days with fever, tachypnea, hypoxemia, and supplemental oxygen. Furthermore, elevated rates of mortality, lower rates of clinical improvement, and lower rates of hospital discharge were observed in patients requiring greater oxygen requirements.

**TABLE 2:** Activin pathway laboratory results and clinical outcomes grouped by baseline supplemental oxygen requirements

	Result for group			
Parameter^*a*^	Low flow (*n* = 62)	High flow (*n* = 135)	Invasive mechanical ventilation (*n* = 116)	*P*
Activin pathway				
Activin A (pg/ml), *n* = 312	236.5 (182.2–346.1)	403.7 (271.7–607.5)	499.6 (324.9–726.9)	<0.0001
Activin B (pg/ml), *n* = 142	67.0 (46.2–81.9)	105.1 (55.9–175.5)	134.0 (74.1–205.4)	0.0008
FLRG (pg/ml), *n* = 301	9399 (7233–12497)	14117 (9889–18800)	15424 (11239–22936)	<0.0001
PAI-1 (ng/ml), *n* = 256	16.7 (12.7–18.6)	17.5 (13.7–22.5)	19.2 (15.5–25.6)	0.004
Clinical outcomes^*b*^				
All-cause mortality	7 (11.3)	33 (24.4)	45 (38.8)	<0.0001
Clinical score improvement (1 point)	50 (80.6)	89 (65.9)	58 (50.0)	0.0001
Improvement in oxygenation	43 (69.4)	93 (68.9)	65 (56.0)	0.06
Discharge	49 (79.0)	84 (62.2)	46 (39.7)	<0.0001
No. of days with fever^*c*^	1 (1–2)	1 (1–4)	4 (1–7)	0.0002
No. of days with tachypnea^*c*^	1 (1–5.3)	2.5 (1–7.8)	6 (3–12.5)	0.0007
No. of days with hypoxemia^*c*^	7 (4.3–10)	12 (8–20)	19 (13–32)	<0.0001
No. of days on supplemental O_2_^*c*^	6.5 (4–10)	11.5 (8–19)	19 (10.5–30)	<0.0001

a Summary statistics are presented as median (IQR) for continuous variables and count (percent) for categorical variables. (A to D) Activin A (A), activin B (B), FLRG (C), and PAI-1 (D).

b One patient that was randomized but not dosed was excluded from this analysis.

c Variables assessed only in survivors.

### Lower activin A, activin B, and FLRG are associated with lower rates of mortality and greater rates of 1-point improvement in clinical score.

**FIG 3:**
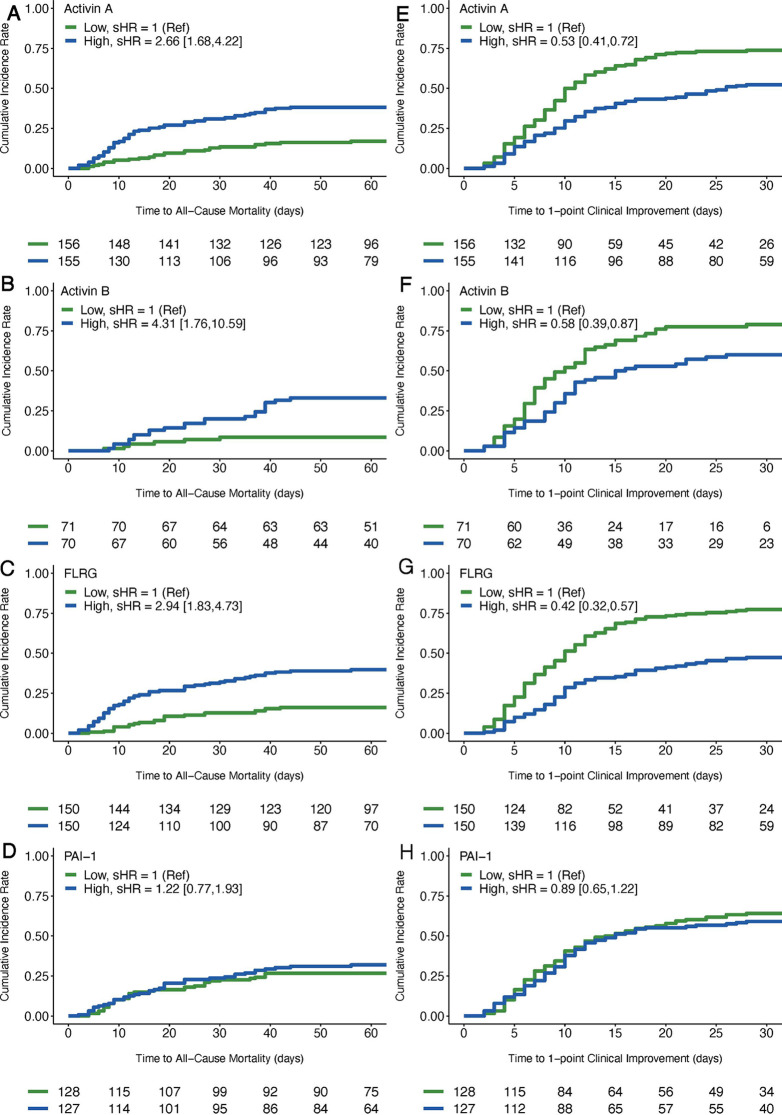
Cumulative incidence curves for all-cause mortality and 1-point clinical score improvement. All biomarkers (activin A, activin B, FLRG, and PAI-1) were median split to form high and low groups. The low biomarker group is shown in green, and the high biomarker group is shown in blue. (A to D) Cumulative incidence rate of all-cause mortality is plotted over time through study day 60. (E to H) Cumulative incidence rate of 1-point clinical score improvement is plotted over time through study day 30. Number of subjects remaining at risk for each event are shown below each plot at 5-day or 10-day intervals starting at study enrollment.

We next assessed the effect of baseline activin A, activin B, FLRG, and PAI-1 on clinical endpoints, including all-cause mortality and clinical score improvement (≥1 point), using Fine-Gray subdistribution hazard models. Patients were divided into two groups based on the median concentrations (low, high) for each analyte (activin A = 389.5 pg/ml, activin B = 88.4 pg/ml, FLRG = 13554 pg/ml, PAI-1 = 17.7 ng/ml). Cumulative incidence curves for both endpoints are shown in Fig. 3 and subdistribution hazard ratios (sHR) are shown in Table 3.

**TABLE 3:** Fine-Gray subdistribution hazard model results

	Result for^*a*^:							
	Activin A (median = 389.5 pg/ml)		Activin B (median = 88.4 pg/ml)		FLRG (median = 13554 pg/ml)		PAI-1 (median = 17.7 ng/ml)	
Parameter	Low	High	Low	High	Low	High	Low	High
No. of patients^*b*^	156	155	71	70	150	150	128	127
All-cause mortality								
No. of events	26 (17%)	59 (38%)	6 (8%)	23 (33%)	24 (16%)	59 (39%)	34 (27%)	40 (31%)
Person-days	8,284	6,821	4,021	3,462	8,072	6,452	6,295	6,014
Event rate^*c*^	3.14 (2.10, 4.60)	8.65 (6.64, 11.08)	1.49 (0.60–3.10)	6.64 (4.31–9.81)	2.97 (1.95–4.36)	9.14 (7.02–11.71)	5.40 (3.80–7.46)	6.65 (4.82–8.97)
Unadjusted sHR	1 (Ref)	2.66 (1.68, 4.22)	1 (Ref)	4.31 (1.76–10.59)	1 (Ref)	2.94 (1.83–4.73)	1 (Ref)	1.22 (0.77–1.93)
Adjusted sHR	1 (Ref)	2.89 (1.71, 4.89)	1 (Ref)	5.59 (1.76–17.76)	1 (Ref)	2.98 (1.67–5.32)	1 (Ref)	0.98 (0.57–1.68)
Clinical score improvement								
No. of events	115 (74%)	81 (52%)	56 (79%)	42 (60%)	116 (77%)	71 (47%)	82 (64%)	75 (59%)
Person-days	3,056	4,882	1,131	1,962	2,792	4,914	3,230	3,482
Event rate^*d*^	3.76 (3.12–4.50)	1.66 (1.33–2.05)	4.95 (3.78–6.38)	2.14 (1.56–2.87)	4.16 (3.45–4.97)	1.45 (1.14–1.81)	2.54 (2.03–3.14)	2.15 (1.71–2.69)
Unadjusted sHR	1 (Ref)	0.53 (0.41–0.72)	1 (Ref)	0.58 (0.39–0.87)	1 (Ref)	0.42 (0.32–0.57)	1 (Ref)	0.89 (0.65–1.22)
Adjusted sHR	1 (Ref)	0.54 (0.40–0.74)	1 (Ref)	0.62 (0.39–0.99)	1 (Ref)	0.46 (0.33–0.64)	1 (Ref)	0.95 (0.67–1.35)

a The study sample was median split for each analyte separately and subdistribution hazard ratios (sHR) along with 95% confidence intervals were calculated for high relative to low groupings. sHR > 1 indicates higher rates in patients with elevated activin A, activin B, FLRG, or PAI-1. sHR < 1 indicates lower rates in patients with elevated activin A, activin B, FLRG, or PAI-1. The variables in the adjusted models include age, sex, race, ethnicity, steroid use, duration of pneumonia prior to baseline, BMI, diabetes, hypertension, and treatment arm as covariates.

b One patient that was randomized but not dosed was excluded from this analysis.

c Per 1,000 person-days.

d Per 100 person-days.

COVID-19 patients with elevated activin A were more likely to die than patients with low activin A (sHR = 2.66, 95% CI = 1.68 to 4.22, *P* < 0.0001). A similar effect was observed for activin B (sHR = 4.31, 95% CI = 1.76 to 10.59, *P* = 0.001) and FLRG (sHR = 2.94, 95% CI = 1.83 to 4.73, *P* < 0.0001). However, no differences in mortality were observed for PAI-1 (sHR = 1.22, 95% CI = 0.77 to 1.93, *P* = 0.40). Further, we did not find statistically significant differences in activin/FLRG/PAI-1 between critical patients on steroids and that that were not on steroids (data not shown).

COVID-19 patients with elevated activin A were less likely to achieve clinical score improvement (≥1 point) than patients with low activin A (sHR = 0.54, 95% CI = 0.41 to 0.72, *P* < 0.0001). A similar effect was observed for activin B (sHR = 0.58, 95% CI = 0.39 to 0.87, *P* = 0.008) and FLRG (sHR = 0.42, 95% CI = 0.31 to 0.57, *P* < 0.0001). However, no differences in clinical improvement were observed based on a median split of PAI-1 (sHR = 0.89, 95% CI = 0.65 to 1.22, *P* = 0.47).

These data demonstrate that activin A/B and their pathway marker FLRG were elevated in the most severe settings of COVID-19 and that high levels were predictive of the worst COVID-19 outcomes; we therefore were interested in further investigating the relationship between the cytokines elevated in the COVID-19 “cytokine storm” and activin A pathway. We studied cell types of particular interest given the pathology of COVID-19bronchial and pulmonary smooth muscle cells.

### IL-1α and TNF-α induce activin A and FLRG in human bronchial/tracheal smooth muscle cells, lung fibroblasts, and pulmonary artery smooth muscle cells.

**FIG 4:**
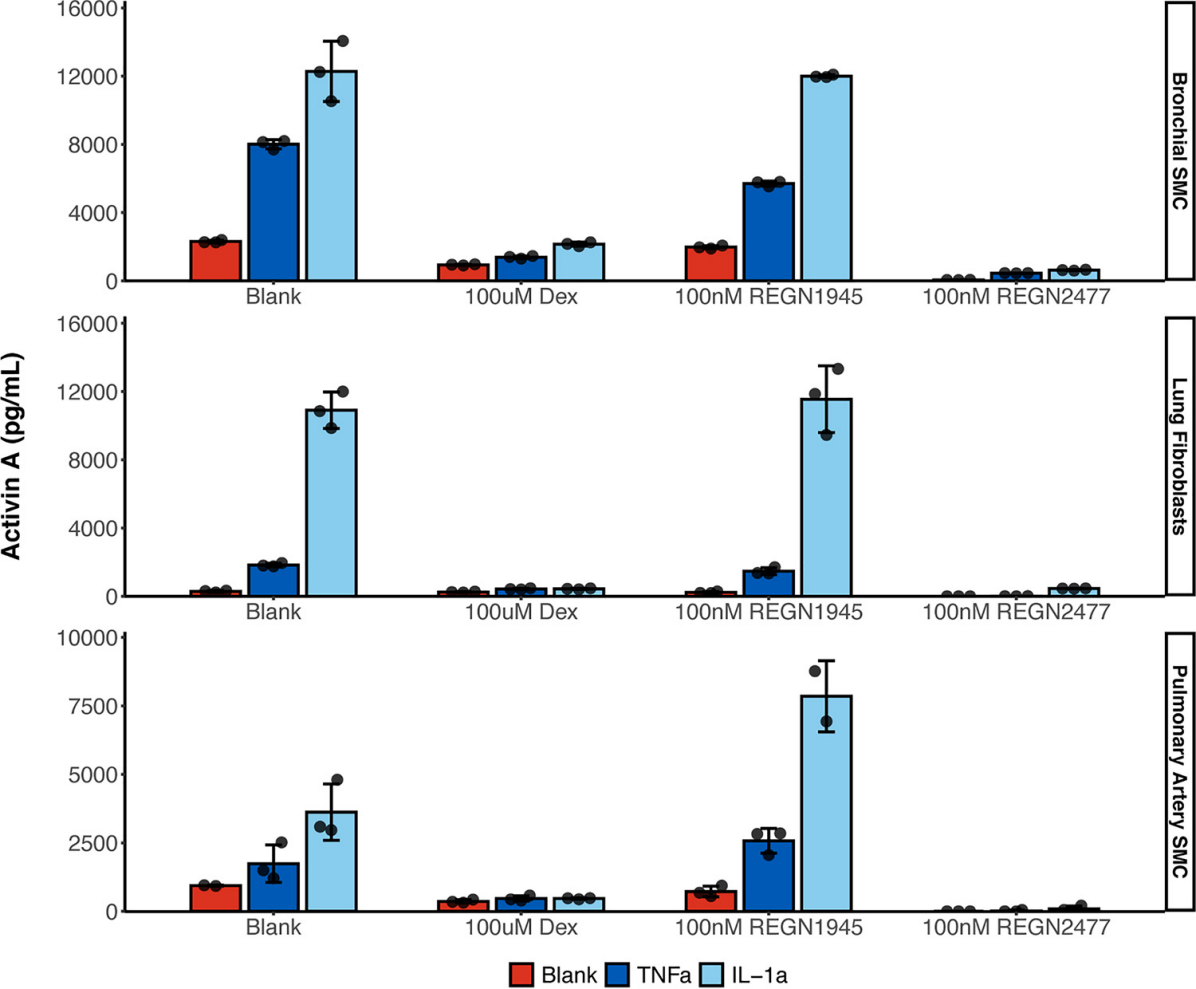
Both IL-1α and TNF-α can stimulate activin A production in bronchial/tracheal SMCs, lung fibroblasts and pulmonary artery SMCs, which can be rescued by dexamethasone. Activin A production was measured in conditioned medium by enzyme-linked immunosorbent assay (ELISA). Bronchial/tracheal smooth muscle (BTSMC) cells, normal human lung fibroblasts (NHLF), and pulmonary artery smooth muscle cells (PASMC) were serum starved overnight and then treated with 10 ng/ml IL-1 α or TNF-α for 5 days in the presence or absence of 100 μM dexamethasone, 100 nM anti-activin A antibody REGN2477 or isotype control REGN1945. Conditioned medium was diluted at 1:10 for ELISA. The experiment was repeated twice, with a consistent result.

We next sought to investigate cells that are relevant for COVID-19 and decided upon studying bronchial/tracheal smooth muscle cells and pulmonary artery smooth muscle cells, to determine if these produced activin A in response to inflammatory cytokines. These cells seemed especially relevant since a prior study noted very high levels of activin A in the bronchial alveolar lavage fluid of ARDS patients (4). A third cell line that we thought to test were bronchial fibroblasts. We included these because it has been shown that COVID-19 is more severe in the aged, and those with preexisting conditions such as COPD (6, 7), and fibroblasts are more prevalent in these settings. These cell types are vulnerable or culprit cell populations in numerous pulmonary diseases— for example, they are contributors to pulmonary remodeling and functional decline in chronic inflammatory lung diseases. Thus, human bronchial/tracheal smooth muscle cells (SMCs), lung fibroblasts and pulmonary artery SMCs were treated with 10 ng/ml IL-1α and TNF-α for 5 days (Fig. 4). We found that both IL-1α and TNF-α stimulated activin A production in bronchial/tracheal SMC, pulmonary artery SMCs and lung fibroblasts but IL-1α had a more potent effect on activin A production in comparison to TNF-α (Fig. 4B). Dexamethasone, a corticosteroid used in a wide range of conditions for its anti-inflammatory and immunosuppressant effect, has been tested in hospitalized COVID-19 patients and found to have benefits for critically ill patients. Here, we also demonstrated that dexamethasone could reduce activin A levels induced by IL-1α and TNF-α back to baseline in bronchial/tracheal, pulmonary artery SMCs, and lung fibroblasts (Fig. 4). Although we used a higher dose (100 μM) of dexamethasone in our *in vitro* study than can be used in the clinic, it is clear that dexamethasone at least is capable of inhibiting cytokine-induced induction of activin A.

To see the effect of anti-activin A, bronchial/tracheal and pulmonary artery SMC as well as lung fibroblasts were treated with an anti-activin A ab (REGN2477) at 100 nM, prior to the treatment with IL-1α or TNF-α. Compared with the isotype control antibody (REGN1945), REGN2477 significantly reduced activin A detection following treatment with IL-1α or TNF-α, indicating that the antibody binds activin A.

### IL-1α and TNF-α induce activin A via the IKK/NF-κB pathway.

**FIG 5:**
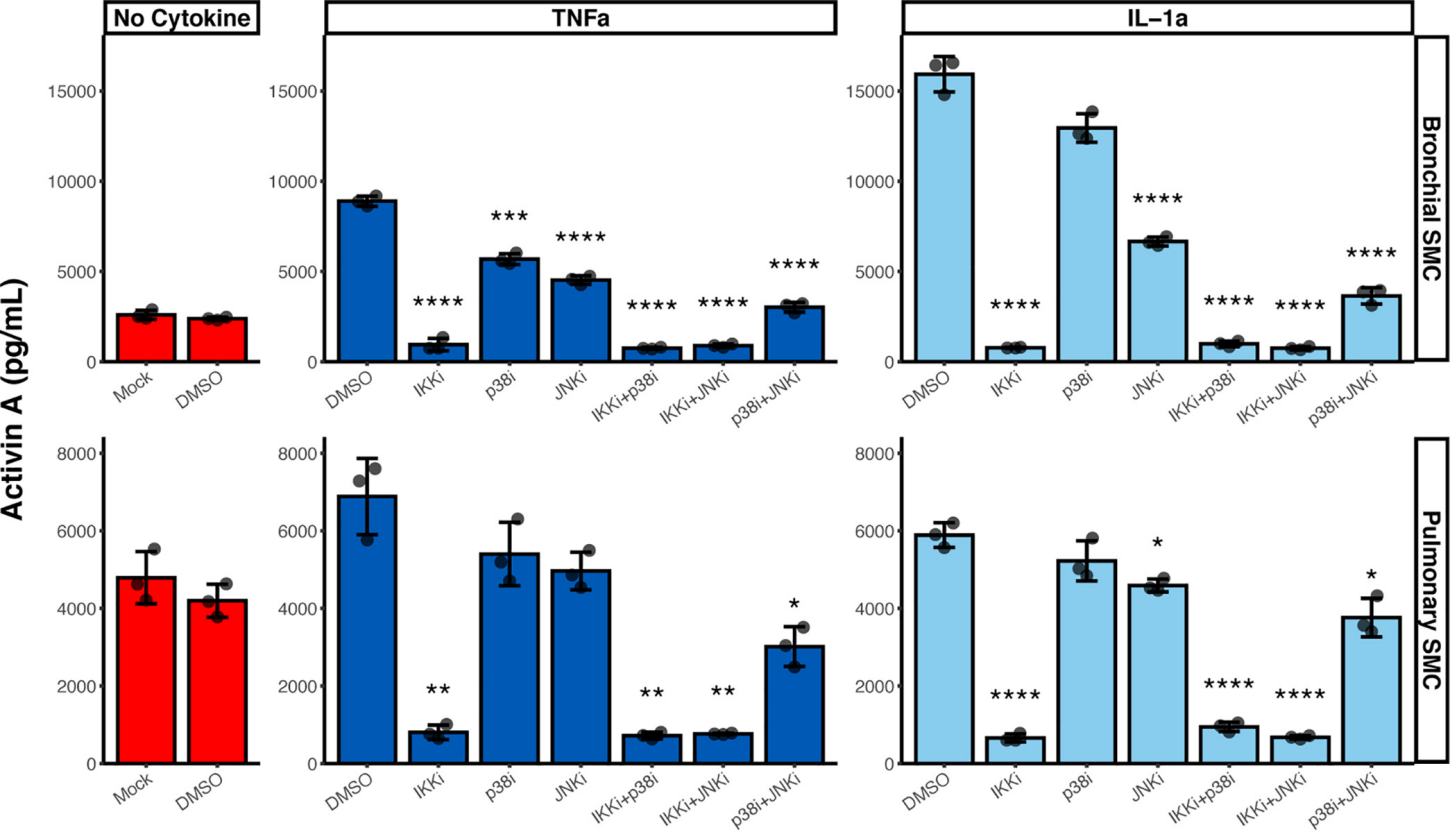
Role of IKK, p38 and JNK in activin A induction in response to cytokine treatment. Bronchial/tracheal smooth muscle cells (SMC) (top) pulmonary artery SMC (bottom) were treated for 24 h with 100 ng/ml of TNF-α or IL-1α in combination with pharmacological inhibitors for IKK (3 μM withaferin A), p38 (0.3 μM SB203580) and JNK (30 μM SP600125). A combination of IKK and p38 inhibitors (same concentrations), a combination of IKK and JNK inhibitors (same concentrations) and a combination of p38 and JNK inhibitors (same concentrations) was also used. DMSO was used as a negative control for inhibitor treatments. Treatment with sterile water only (Mock) was used as an additional control. Activin A levels were quantified in conditioned media by ELISA. *n* = 3 for each treatment. Significant Bonferroni-corrected pairwise comparisons with DMSO within each treatment condition are labeled. *, *P* < 0.05, **, *P* < 0.01, ***, *P* < 0.001, ****, *P* < 0.0001.

We next wanted to further explore the mechanism by which inflammatory cytokines can induce activin A, in pulmonary artery and in bronchial smooth muscle cells, which are both relevant for COVID-19 pathology—since SARS-Cov2 prominently effects the lung and the blood vessels (Fig. 5). In particular, we wanted to dissect the role of signaling molecules IKK, p38 and JNK that are induced in response to cytokine receptor activation.

In each cell type, the inflammatory cytokine mediated production of activin A was blocked by inhibiting the IKK/NFkappaB pathway (Fig. 5). Inhibition of p38 or JNK could partially inhibit activin A production, but this was cell-context specific, and not at all as complete as simply blocking IKK (Fig. 5). Furthermore, simultaneous inhibition of IKK and p38 or IKK and JNK was not additive, since IKK inhibition was sufficient to almost fully inhibit activin A production (Fig. 5).

These results indicate that activin A induction by IL-1α and TNF-α is mainly dependent on the IKK/NF-κB pathway and can be partially inhibited by blocking p38 or JNK pathways.

### Blockade of activin A in SARS-Cov2-infected hamsters does not increase viral load and shows a hint of benefit.

**FIG 6:**
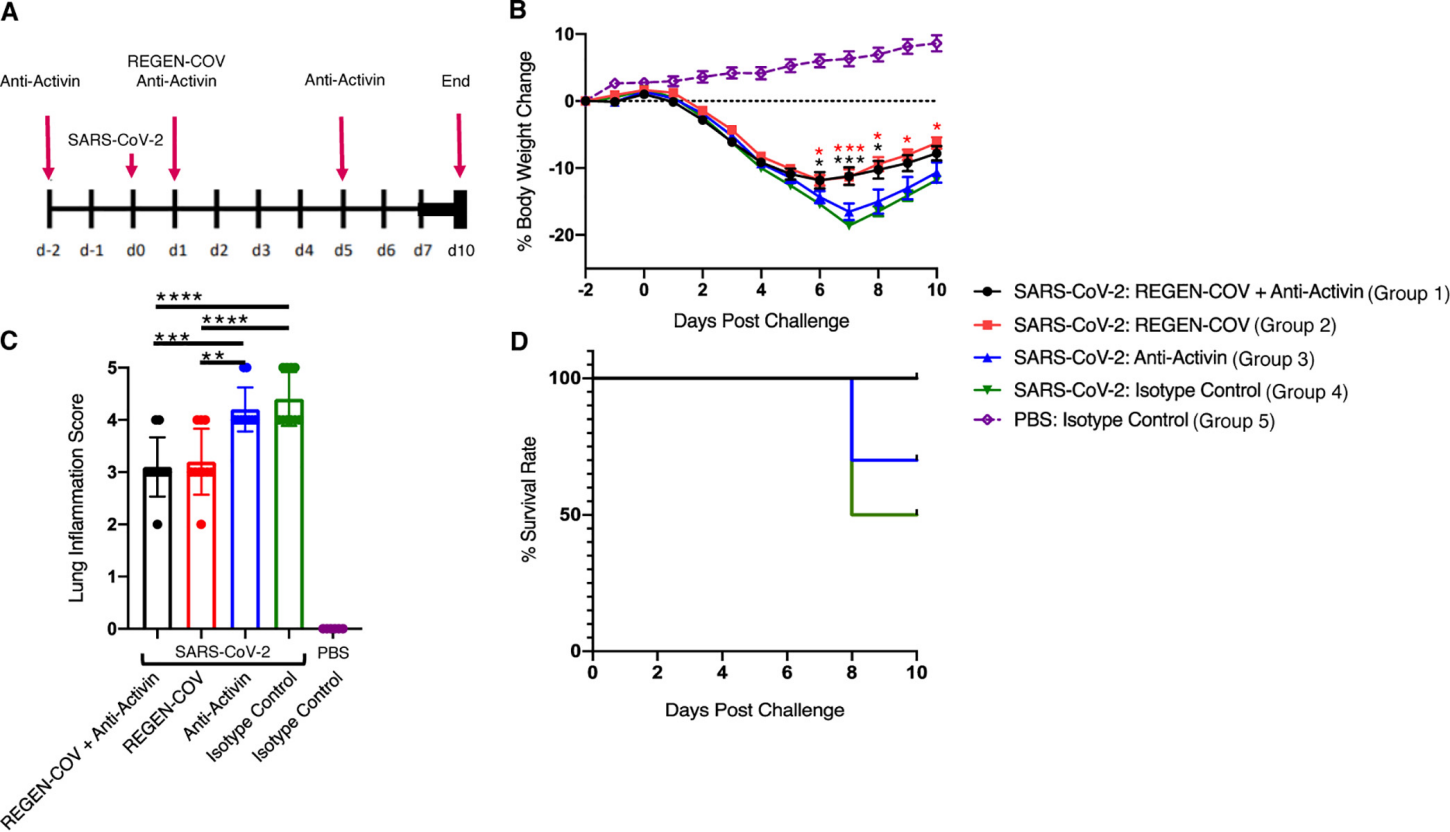
Efficacy of the anti-activin A antibody alone, REGEN-COV antibody cocktail alone, and the combination of the anti-activin antibody and REGEN-COV cocktail in the golden Syrian hamster model of SARS-CoV-2. (A) Study design overview and the antibody administration schedule. SARS-CoV-2 challenge was administered to groups 1 to 4 on day 0 (*n* = 10 per group). Group 5 received PBS and served as a healthy control (*n* = 6). (B) Daily body weight changes; (C) lung inflammation grading scores; (D) survival curves. In panel B, data are mean ± standard error of mean (SER). The *y* axis represents percent body weight change from the baseline, 2 days (day -2) prior to SARS-CoV-2 challenge. *x* axis represents days prior and postchallenge. Black asterisks indicate mean body weight differences between groups 1 (black line) and 4 (green line). Red asterisk indicates mean body weight difference between Groups 2 (red line) and 4 (green line). In panel C, the *y* axis represents semiquantitative scores for lung inflammation. Data are mean ± standard deviation from the mean (SD). Asterisks indicate difference between SARS-CoV-2 challenged groups. In panel D, the *y* axis represents percentage of surviving hamsters. *x* axis represents days following SARS-CoV-2 challenge. Statistical analyses were conducted with a one-way ANOVA followed by Tuckey’s multiple-comparison tests. SARS-COV-2 challenged groups were compared. *, *P* < 0.05; **, *P* < 0.01; ***, *P* < 0.001; ****, *P* < 0.0001.

The hamster model of SARS-CoV2 infection has been used to test anti-Spike protein antibodies (13), along with other potential treatment modalities (14). To evaluate the efficacy of blocking activin A, we treated SARS-CoV-2 infected hamsters with a combination of REGEN-COV cocktail and the anti-activin A antibody, REGEN-COV cocktail alone and the anti-activin A antibody alone (Fig. 6A). Here, the anti-activin A antibody was used in preventative mode, administration starting 2 days prior to SARS-CoV-2 challenge, while REGEN-COV cocktail was used in treatment mode, administration starting on day 1 following challenge (Fig. 6A). We used a suboptimal dose of REGEN-COV cocktail (5 mg/kg) to achieve only partial protection from the infection. Hamsters were followed for 10 daysfollowing challenge which allowed us to observe worsening of the disease, as well recovery from the disease.

As previously shown, treatment with the low dose of REGEN-COV cocktail (5 mg/kg) was protective and reduced body weight loss in all SARS-CoV-2 challenged hamsters (Fig. 6B). There was no additional benefit of the anti-activin A antibody for the body weight loss prevention (Fig. 6B).

Five out of 10 SARS-CoV-2 challenged hamsters that were treated with the isotype control antibodies lost more than 20% body weight and were euthanized unscheduled on day 7 following challenge (Fig. 6D). Treatment with REGEN-COV cocktail alone or in combination with the anti-activin A antibody was fully protective from severe morbidity (Fig. 6D). Treatment with the anti-activin A antibody alone was partially protective from severe morbidity and in this group, three out of 10 hamsters were euthanized unscheduled on day 7 due to the excessive body weight loss (Fig. 6D). Although difference in the survival between the anti-activin A antibody treated group and the isotype control treated group was not statistically significant (Fig. 6D).

We next performed histopathological examination of lungs collected on day 7 in case of hamsters that were prematurely euthanized due to >20% body weight loss and on day 10 for all surviving hamsters. SARS-CoV-2 induced “marked” to “severe” inflammation in the lungs of hamsters treated with the isotype control antibodies (Fig. 6C; Table 4). Severity of lung inflammation was notably reduced in the groups treated with REGEN-COV cocktail alone or in combination with the anti-activin A antibody (Fig. 6C; Table 4); none of the animals in these groups showed severe lung inflammation. The anti-activin antibody did not impact the severity of SARS-CoV-2 induced lung inflammation (Fig. 6C; Table 4). Finally, there was no enhancement of lung pathology with the anti-activin A antibody, demonstrating that activin-A blockade does not exacerbate lung pathology. In this experiment, we were unable to reliably quantify viral loads, because lung specimens were contaminated during collection and viral RNA was found in many healthy control samples.

**TABLE 4:** Histopathological findings in lungs^*a*^

	Result for group:						
Parameter	1	2	3		4		5
Challenge	SARS-COV-2						PBS
Treatment	REGEN-COV + Anti-activin A	REGEN-COV	Anti-activin A		Isotype controls		
Days postchallenge	10	10	7	10	7	10	10
Analyzed animal #	10	10	3	7	5	5	6
Lung inflammation							
None	0	0	0	0	0	0	6
Minimal	0	0	0	0	0	0	0
Mild	1	1	0	0	0	0	0
Moderate	7	6	0	0	0	0	0
Marked	2	3	2	6	2	4	0
Severe	0	0	1	1	3	1	0

a H&E-stained lung sections were scored for severity of inflammation. Numbers for “Lung inflammation” indicate numbers of affected animals.

**FIG 7:**
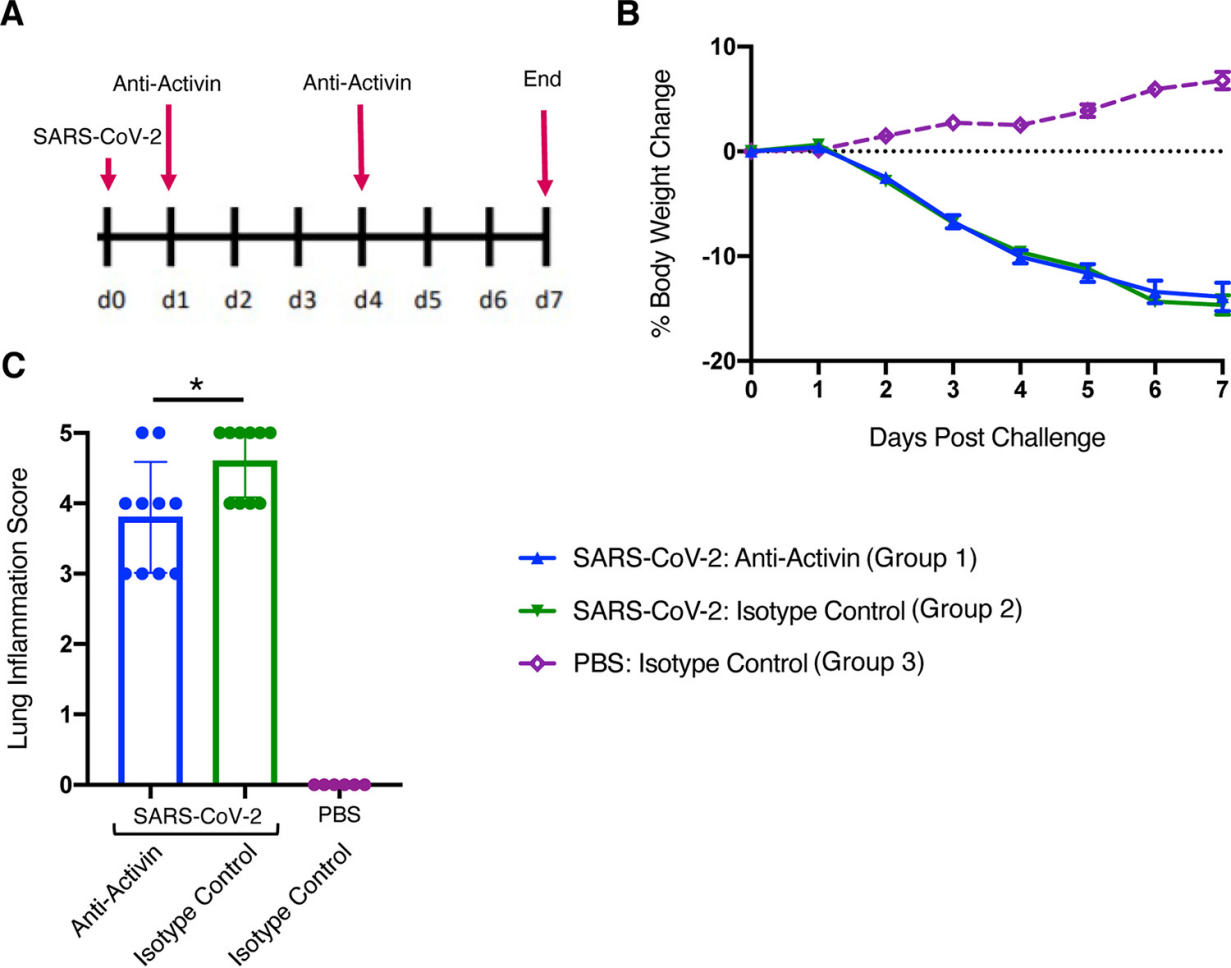
Efficacy of the anti-activin antibody A in the golden Syrian hamster model of SARS-CoV-2. (A) Study design overview and the antibody administration schedule. SARS-CoV-2 challenge was administered to froupls 1 and 2 on day 0 (*n* = 10 per group). At this point, group 3 (*n* = 6) received PBS and served as a healthy control. (B) Daily body weight changes; (C) lung inflammation grading scores. In panel data are mean ± SER. The *y* axis represents percent body weight change from the baseline (day 0), recorded prior to SARS-CoV2 challenge. *x* axis represents days following SARS-CoV2 challenge. In panel B, comparisons among SARS-COV-2 challenged groups were done with a one-way ANOVA followed by Tuckey’s multiple-comparison tests and no differences were found. In panel C, the *y* axis represents semiquantitative scores for lung inflammation. *, *P* < 0.05 indicates difference between SARS-CoV-2 challenged groups derived from a one-way ANOVA followed by Tukey’s multiple-comparison tests. Data are mean ± SD.

**FIG 8:**
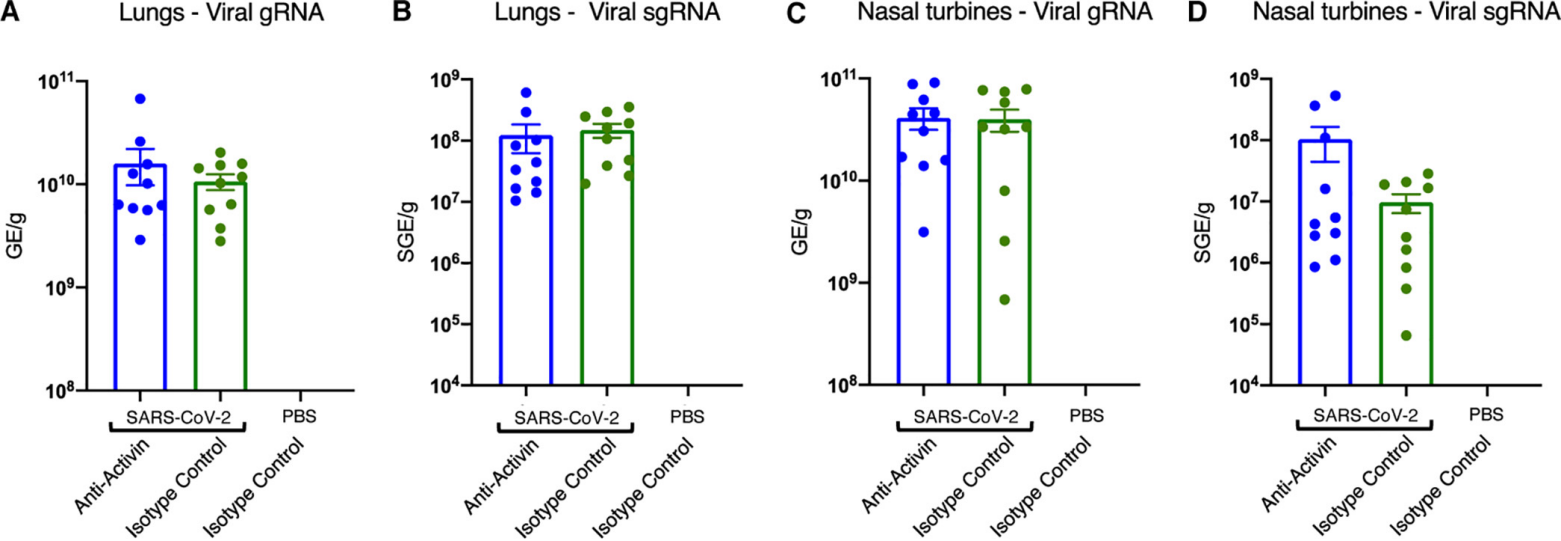
Viral genomic RNA (gRNA) and subgenomic RNA (sgRNA) in lungs and nasal turbines on day 7 following SARS-COV-2 challenge. Comparisons between SARS-COV-2 challenged groups were done with a one-way ANOVA followed by Tuckey’s multiple-comparison tests and no differences were found. Two out of 6 nasal turbine samples from healthy controls were contaminated and tested positive for viral gRNA. *y* axes are set to log_10_ scale and represent genomic equivalents (GE) per gram. Data are mean ± SER.

To address the potential effect of activin A blockade on viral load, we measured viral loads in another experiment, where we treated SARS-CoV-2 infected hamsters with the anti-activin A antibody for 7 days following challenge (Fig. 7A). Similar to the first experiment, treatment with the anti-activin A antibody did not protect hamsters from SARS-CoV-2 induced body weight loss (Fig. 7B), although histological examination of lung inflammation showed a slight benefit of blocking activin A (Fig. 7C). All lungs (10/10) collected from the isotype control treated group showed “marked” to “severe” inflammation on day 7 following challenge (Fig. 7C), while four out of 10 hamsters treated with anti-activin A antibody showed “moderate” inflammation (Fig. 7C). Importantly, we did not observe increased viral load with the anti-activin A antibody treatment (Fig. 8A to D).

## DISCUSSION

COVID-19 is a recent and potentially very serious disease; in the most severe settings it induces acute respiratory disease syndrome (ARDS), which can lead to the need for mechanical ventilation (15). Severe cases of COVID-19 can also result in death; worldwide mortality from the disease ranges from 1.5 to 15%, depending on the country and other clinical risk factors. ARDS can be caused by a variety of other conditions, including sepsis (16, 17). In a prior survey of non-COVID-19 ARDS patients, it was noted that there was a significant increase in levels of activin A in the bronchial alveolar lavage fluid relative to control levels (4); these same researchers demonstrated that activin A was sufficient to induce an ARDS-like phenotype in a preclinical model, by delivering the gene intratracheally via an adenovirus (4). A follow-up to this study was able to reproduce the prior finding and demonstrated that activin A and its pathway marker, FLRG (follistatin-like related gene, also called FLSTL3), were demonstrably elevated in the sera of non-COVID-19 ARDS patients (5). Such studies are clearly needed, to establish reliable biomarkers the reproducibility of biomarkers for ARDS; what is especially needed are biomarkers which predict disease severity so that clinicians can be alerted to the need for stronger interventions.

When patients with severe ARDS from COVID-19 began to be studied it was reported early on that these patients were experiencing a “cytokine storm,” which included reports of many inflammatory cytokines, including IL-1 and TNF-α upregulated in the blood. Our group had previously connected IL-1 and TNF-α to the induction of activin A, in a study of skeletal muscle atrophy caused by these cytokines (3). We were interested in the mechanism of inflammation-induced atrophy, since inflammatory cytokines are upregulated in several settings of cachexia, including cancer cachexia, and the resultant loss of muscle is a serious contributor to mortality (18). In our prior study we found that blocking activin A could inhibit approximately 50% of the cytokine-induced muscle atrophy, demonstrating also in that setting that activin A had a causal role for the downstream effects of inflammatory cytokines (3). In the lung ARDS study, it was also shown that inhibiting the activin A pathway with a receptor trap could block the ability of LPS to induce ARDS (4), further strengthening the evidence that activin A was required to induce ARDS, at least in a preclinical model.

We therefore sought in the present study to determine if activin A and B and their pathway marker, FLRG, were upregulated in patients with COVID-19, and especially those who had ARDS, in comparison to age-matched controls. In this study, we found that both activin A, activin B, and FLRG were upregulated quite significantly in ICU-bound COVID-19 patients, with some patients experiencing more than 2-fold increases in activin A, with a tail of subjects that experienced increases as high as 8× above the normal median. Strikingly, activin A levels were not significantly increased in COVID-19 patients who had serious disease but did not require invasive mechanical ventilation; the marker clearly distinguished the populations. activin A induces FLRG (follistatin-related gene, also known as follistatin-like 3) (19). In contrast to activin A, which is internalized—and thus may not be found in the blood at lower levels of pathway activation—FLRG remains in the bloodstream, where it inhibits activin function; thus it is a downstream marker of prior activation of the pathway. FLRG levels were induced in the severe, non-ICU patients, but were even more elevated in the ICU patients, especially those who were experiencing ARDS, as determined by their need for invasive mechanical ventilation. Additionally, with FLRG, subjects with very high elevations were found to be more likely to require invasive mechanical ventilation as well.

**FIG 9:**
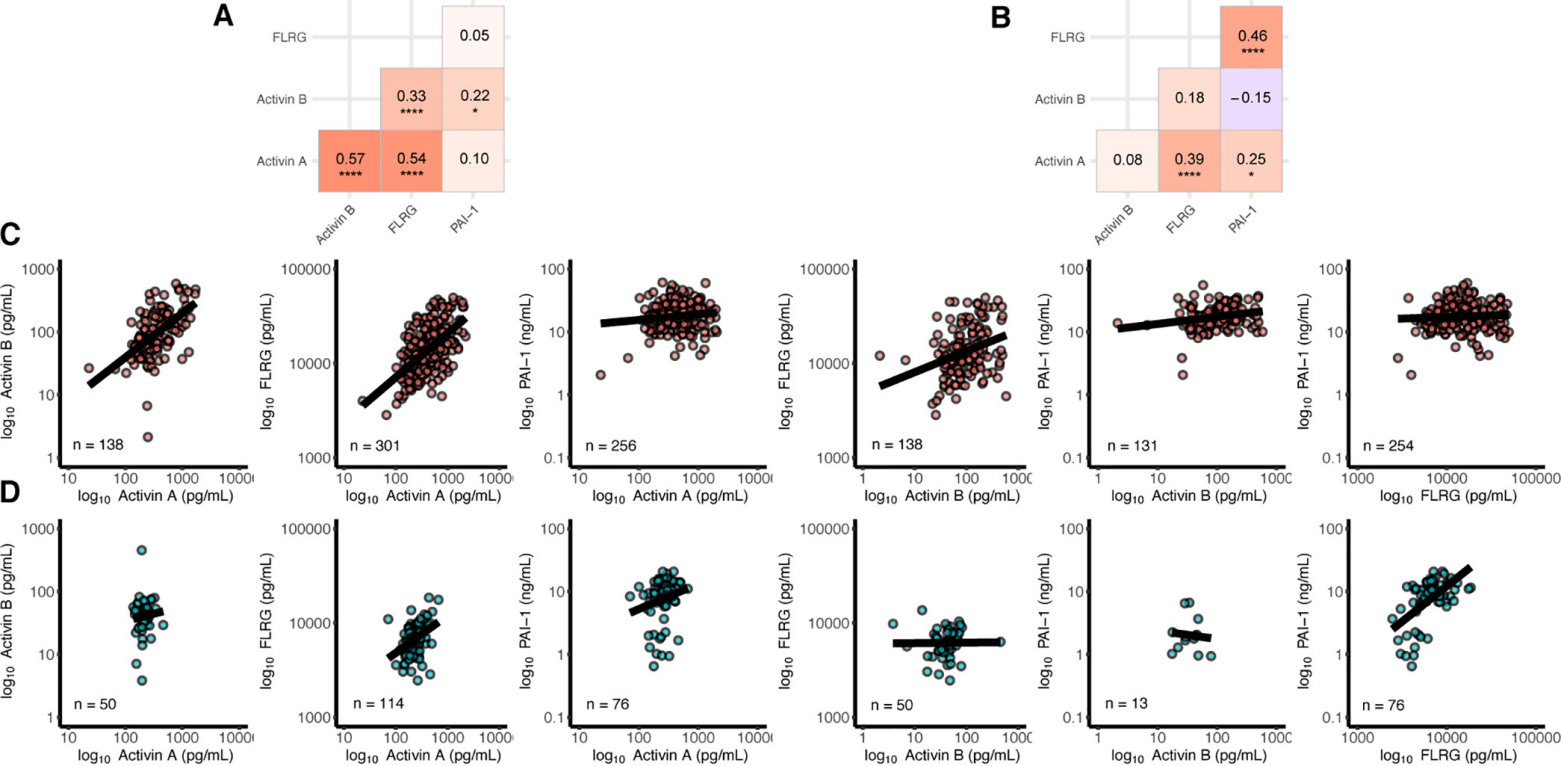
Moderate positive correlations observed between activin A, activin B, and FLRG in COVID-19 patients (A and B). Correlation matrices for COVID-19 patients prior to dosing (A) and control subjects (B). Spearman rho correlation coefficients are shown, *, *P* < 0.05, **, *P* < 0.01, ***, *P* < 0.001, ****, *P* < 0.0001. (C and D) Scatterplots of each biomarker comparison from COVID-19 patients prior to dosing (C) and control subjects (D). For visualization purposes all analytes were log_10_ transformed.

The action of activin A and FLRG is likely not to be independent, with a correlation of (Spearman rho = 0.57, *P* < 0.0001) (Fig. 9) at baseline between the two analytes, providing statistical evidence that they're likely within the same pathway, which has been demonstrated biologically (15). In contrast, PAI-1, which is a marker of coagulation, is already elevated even in less severely affected patients, and was not further elevated in the ICU patients. Therefore, the data in this paper establishes that activin/FLRG levels distinguish those who go on to the most serious form of the disease, as determined by the need for oxygen, and risk of dying from COVID-19. In contrast, PAI-1 is a less reliable biomarker for the eventual need for oxygenation, or the risk of mortality. Although the data does not itself provide evidence that elevated activin/FLRG are causative of morbidly and mortality, it does indicate that these are appropriate targets for further analyses, and prior preclinical data were suggestive that activin A may be causative for ARDS (4). In the present analysis, baseline levels of activin A and FLRG (not PAI-1) predicted all-cause mortality both with and without inclusion of covariates in the models. A one standard deviation difference in activin A (+374.2 pg/ml) at baseline resulted in a 54% increased likelihood of death and a one standard deviation difference in FLRG (+9413 pg/ml) resulted in a 68% increased likelihood of death. In a complementary analysis, patients with activin A greater than the sample median (389.5 pg/ml) were 2.7 times more likely to die than patients with lower levels. Patients with FLRG greater than the sample median (13554 pg/ml) were 2.9 times more likely to die than patients with lower levels. These effects remained consistent when treatment arm and other covariates were included in the models. Similar trends were observed for activin B, but the sample size available for this analysis were likely not large enough.

Prior studies have shown that activin A can cause fibrosis (20), which is seen in ARDS lungs, perhaps this is one way in which activin A contributes to the phenotype. Further, activation of myostatin, a related ligand, was shown to block skeletal muscle differentiation (21); however, these transforming growth factor β superfamily molecules can induce fibroblast proliferation (22)—which is also suggestive of their possible role in this clinical setting. In addition, a paper looking at age-associated changes in protein levels in human plasma was recently published (23); when we analyzed activin A (InhbA.InhbB) and FLRG (FSTL3) using their publicly available data (https://twc-stanford.shinyapps.io/aging_plasma_proteome/), both were shown to be significantly upregulated with age, and this upregulation could lower the threshold for pathological upregulation induced by inflammatory cytokines.

We were interested to explore the mechanism by which inflammatory cytokines induce activin A, and what the likely cell types were which might be responsible. Examination of cell lines demonstrated that IL-1 could induce activin A significantly in bronchial smooth muscle cells, as well as pulmonary muscle cells. It also had this effect on lung fibroblasts, which might be more prevalent in the aged, or those with preexisting conditions, such as COPD. TNF-α was a less reliable inducer of activin A in fibroblast cell lines; we speculate that this may be due to relative levels of IL-1 versus TNF receptors, rather than some intrinsic difference in the signaling pathways; we note this because TNF-α is fully capable of inducing activin A in pulmonary and bronchial smooth muscle cells, so there does not seem to be anything intrinsically different between the two cytokines, just a cell-specific ability to respond. As to downstream mechanisms responsible for the induction of activin A, we distinguished between various signaling pathway induced by IL-1 and TNF using cellular inhibitors of those pathways. Only the IKK inhibitor was able to completely and consistently block the induction of activin A, as opposed to p38 or Jnk inhibitors, which were less effective. IKK is upstream of NF-κB induction, so the data demonstrate that activin A is induced by this pathway.

The cellular differences in response to IL-1 versus TNF demonstrate that a cytokine-centric approach is unlikely to be successful to treat a disease like COVID-19, where there is a large array of cytokines induced. Many of these induced cytokines signal similarly, and therefore one cannot hope to block the ultimate effect without going downstream in the signaling pathway. Thus, it is of particular interest that activin A is induced by the IKK/NF-κB pathway, in common to IL-1 and TNF, and other inflammatory cytokines, and that activin A is sufficient, at least in a preclinical model to induce ARDS. Furthermore, since activin A and its pathway marker, FLRG, are unique in being associated with the most severe effects caused by COVID-19 (the induction of ARDS, resulting in an inability to breathe, ultimately causing death), the strong suggestion from these data is that it would be beneficial to COVID-19 patients suffering from ARDS to be treated with an inhibitor of activin A.

Of note, an antibody to activin A was fully capable of blocking the measured activin A levels induced by inflammatory cytokines, giving further hope that such an antibody could be effective in blocking the severe effects of COVID-19 in ICU patients. Indeed, in the previously published preclinical setting, use of an activin receptor trap was sufficient to ameliorate ARDS in an LPS-induced model of the syndrome (4).

While we were preparing our manuscript, another manuscript appeared similarly demonstrating activin A was disregulated in COVID-19 patients (11). The findings in the present manuscript are in agreement with that paper. In addition, we extended the observations to demonstrate elevation of the activin pathway according to supplemental oxygen requirements and clinical improvement based on reduction of supplemental oxygen requirements. Further, we provide a mechanism by which activin is upregulated, showing that cytokines that activate the NF-kappa B pathway can induce activin A in fibroblasts and bronchial smooth muscle. The current manuscript is also unique in that we sought to ask if there might there be a benefit or any negative consequences to using an anti-activin A approach in ARDS patients using a preclinical model of COVID-19 infection. This is particularly important because one study did show that activin A could inhibit viral production in a variety of virus-infected cell lines (24), therefore we were curious to evaluate if blockade of the pathway can lead to increased virus load and exacerbation of disease. This seems to be a plausible evolutionary mechanism explaining why activin A might be induced by cytokines. Initial viral load does correlate with disease severity (25). However, the cytokine storm often comes later in the disease, indicating the cytokine storm is part of the immune response to the virus (26). The impression, therefore, is that these severely affected patients are not suffering directly from the viral load, but instead from an overreaction of the immune system—the later occurring cytokine response, which in some patients overinduces activin A. Nevertheless, we performed intervention studies in a preclinical model of COVID-19—the hamster model, which is the most commonly used nonprimate model for testing interventions against the SARS-CoV-2 virus (13, 14, 27). In the context of testing therapies for blocking activin A, the shortcomings of this model should be pointed out: the hamster model is acute (7 to 10 days following the infection), and the studies conducted are in young animals. In contrast, the risk of severe COVID-19 in humans goes up significantly with age, as does evidence of activin signaling, and is especially prevalent in aged patients with comorbidities; thus the hamster model is perhaps most useful for derisking activin therapy, as opposed to predicting its efficacy.

In this model, no apparent increase in SARS-Cov-2 viral loads and lung pathology was seen upon treatment with the anti-activin A antibody, which helps in derisking this concern with the treatment. In addition, there was a slight benefit of the anti-activin A antibody treatment alone. In one experiment, there was less mortality with the anti-activin A antibody versus the negative control, although this did not reach statistical significance. In the other experiment, some improvement in lung pathology was observed in the anti-activin A antibody treated group versus the negative control, although this result did not reproduce across the two studies. No apparent increase in SARS-Cov2 viral loads and lung pathology was seen upon treatment with the anti-activin A antibody, which helps in de-risking this concern with the treatment.

We therefore suggest further exploration of activin A inhibition in the treatment of COVID-19 patients with ARDS.

## MATERIALS AND METHODS

### Clinical study. (i) COVID-19 samples and informed consent.

Samples were collected from subjects who consented to participate in an adaptive, phase 2/3, randomized, double-blind, placebo-controlled trial of intravenous (IV) sarilumab in adults hospitalized with severe or critical COVID-19. In the phase 3 portion, patients with critical COVID-19 were randomized to sarilumab 400 mg IV, sarilumab 200 mg IV, or placebo (trial registration number: NCT04315298). The protocol was developed by the sponsor (Regeneron Pharmaceuticals Inc.). Clinical data were collected by the study site investigators and analyzed by the sponsors. The local institutional review board or ethics committee at each study center oversaw trial conduct and documentation. All patients provided written informed consent before participating in the trial.

### (ii) Definition of clinical variables.

Clinical variables were defined as follows. For oxygen device type, high-flow oxygen requirements include nonrebreather face mask, high-flow nasal cannula, noninvasive ventilation, and low-flow oxygen requirements include nasal cannula, simple face mask. All-cause mortality is the number of days from study randomization to death. Clinical improvement is ≥1-point improvement in clinical status from baseline to day 29 using the clinical status assessment (7-point ordinal scale) (28): 1, death; 2, hospitalized, requiring invasive mechanical ventilation membrane; 3, hospitalized, requiring noninvasive ventilation or high-flow oxygen devices; 4, hospitalized, requiring supplemental oxygen; 5, hospitalized, not requiring supplemental oxygen but requiring ongoing medical care (COVID-19 related or otherwise); 6, hospitalized, not requiring supplemental oxygen and no longer requiring ongoing medical care; and 7, not hospitalized.

### (iii) Statistical analysis.

Descriptive statistics grouped by disease severity and oxygenation requirements are reported as median (interquartile range; IQR) for continuous variables and frequency (percent) for categorical variables. Kruskal Wallis tests were used for continuous variables across three or more groups with follow-up Dunn pairwise comparisons. Wilcoxon signed-rank tests were used for continuous variables between two groups. Fisher’s exact tests were used for categorical variables. For longitudinal outcomes, logistic regression was used with baseline activin A, activin B, FLRG, and PAI-1 (standardized) as predictors in separate models, with and without inclusion of covariates. Fine-Gray subdistribution hazard models were also generated for these longitudinal outcome variables with available time-to-event information. Subjects were split into two groups (low and high) based on median concentrations of each analyte at baseline. Subdistribution hazard ratios (sHR) for high groups, relative to low groups, were calculated with and without inclusion of covariates. All-cause mortality and clinical score improvement time-to-event data were censored to day 60 and day 29, respectively. A type I error rate of α = 0.05 was used as the threshold for statistical significance, with Bonferroni adjustment for follow-up tests and multiple comparisons. Covariates for all indicated analyses included age, sex, race, ethnicity, steroid use, duration of pneumonia prior to baseline, body mass index (BMI), diabetes, and hypertension. Treatment arm was included as a covariate in models predicting longitudinal outcomes. The results were almost identical regardless of inclusion or exclusion of covariates; therefore, unadjusted statistics are reported in the text. All statistical analyses were performed using R version 3.6.1.

### *In vitro* experiments. (i) Reagents.

Withaferin A (catalog number S8587), SB203580 (catalog number S1076), and SP600125 (catalog number S1460) were acquired from Selleckchem. Recombinant human TNF-α (catalog number 300-01A), IL-1β (catalog number AF-200-01B), and IL-1α (catalog number 200-01A) were acquired from Peprotech.

### (ii) Cytokine and steroid treatments.

Bronchial/tracheal smooth muscle cells (BTSMC; ATCC, PCS-130-011), normal human lung fibroblasts (NHLF; Lonza, CC-2512), and pulmonary artery smooth muscle cells (PASMC; Lonza, CC-2581) were seeded at 200,000 cells/well in 12-well plates and cultured in media as specified by manufacturers. To eliminate the influence of other factors, cells were washed once and cultured with serum-free medium with 5% BSA overnight. To study the effect of proinflammatory cytokines IL-1α and TNF-α on activin A production and whether dexamethasone and anti-activin A can rescue, cells were pretreated with 100 μM dexamethasone (Sigma-Aldrich Inc, #D2915), 100 nM REGN1945 (isotype control) or 100 nM REGN2477 (anti-activin A) for 10 min, and then treated with or without 10 ng/ml IL-1α (R&D Systems, #200-LA-CF) or TNF-α (R&D Systems, #210-TA-CF) for 5 days. Conditioned media were harvested and diluted at 1:10, and ELISA (human/mouse/rat activin A Quantikine ELISA kit, R&D Systems, #DAC00B) was performed to measure activin A production according to the manufacturer’s protocol.

### (iii) ELISAs.

Activin A, FLRG, and PAI-1 ELISAs were carried out by using R&D Quantikine ELISA kit systems, DAC00B, DFLRG0, DSE100, respectively, according to manufacturer’s protocols. The activin B ELISAs from Ansh Labs (catalog no. AL-150) were performed according to the manufacturer’s protocol.

### (iv) Statistical analysis.

***(a)*** **In vitro** ***experiment 1.*** Within each treatment condition (blank, TNF-α, and IL-1α) and cell type (bronchial SMC, lung fibroblasts, and pulmonary artery SMC), pairwise *t* tests were used to compare activin A concentration after additional cotreatment of 100 μM dexamethasone, 100 nM REGN, and 100 nM REGN2477 to no cotreatment (blank).

***(b)*** **In vitro** ***experiment 2.*** Within each main treatment condition (no cytokines, IL-1α, TNF-α) and cell type (bronchial smooth muscle, pulmonary smooth muscle), activin A induction for each cotreatment was compared to DMSO cotreatment using pairwise *t* tests.

### Studies in a hamster SARS-COV-2 model. (i) Antibody formulations for *in vivo* studies.

All antibodies used *in vivo* were developed by Regeneron, formulated in sterile saline and administered intraperitoneally. The anti-activin A antibody and the respective isotype control (IgG4) were administered at 25 mg/kg body weight. REGEN-COV cocktail, which consist of two antibodies REGN10933 and REGN10987 was administered at 5 mg/kg (2.5 mg/kg for each antibody). The isotype control (IgG1) for REGEN-COV cocktail was administered at 5 mg/kg.

**(ii) *In vivo* studies.** The hamster studies were conducted at Bioqual Inc, Rockville, MD, USA, according to BIOQUAL Inc. Institutional Animal Care and Use Committee (IACUC)-approved protocols. Male golden hamsters aged 6 to 8 weeks were housed at the Bioqual animal facility and acclimated for at least 6 days. SARS-CoV-2 challenge was carried out with 2019-nCoV/USA-WA1/2020, BIOQUAL lot number 12152020-1235, Titer: 600,000 (Vero76), 36,875,000 (TMPRSS2) PFU/ml. Hamsters were challenged at 1:10 dilution in 100 μl (6e3 PFU per hamster, Vero E6 derived) intranasally on day 0. At this point, healthy control animals were pseudo-challenged with sterile phosphate-buffered saline (PBS). Antibody treatment schedules are schematically shown in Fig. 6A and 7A.

All animals were weighed and monitored daily. Hamsters, who’s body weight was reduced >20% from the baseline during the *in vivo* portion of the study were euthanized, followed by tissue collection. At the end of experiments (Fig. 6A and 7A), hamsters were euthanized under sedation with isoflurane followed by tissue collection.

### (iii) Tissue collection and processing.

For viral load determination, 0.1 to 0.2 g specimens were collected from right inferior lung lobes and nasal turbines (two specimens per animal), snap-frozen in liquid nitrogen and stored at −80°C for further analysis. Entire left lung lobes were collected in 10% neutral buffered formalin for histopathology. Paraffin embedding, sectioning and hematoxylin and eosin staining of lung specimens was performed at Histoserv Inc., MD, USA. Slides were imaged at ×40 magnification and evaluated by a board-certified veterinary pathologist.

### (iv) Histopathological evaluation of lungs.

The severity of lung inflammation was assessed blindly by board certified veterinary pathologists. The severity of inflammation was graded using a semiquantitative score from 0 to 5, as follows: 0 or none is no inflammation; 1 or minimal is spots of inflammation visible only at high power; 2 or mild is to 5% of lung tissue with inflammation; 3 or moderate is >6 to 33% (1/3rd) of lung tissue with inflammation; 4 or marked is >33 to 66% (two-thirds) of lung tissue with inflammation; 5 or severe is >67% (more than two-thirds) of lung tissue with inflammation.

### (v) Viral load determination.

Viral loads were determined in tissue specimens from lungs and nasal turbines. SARS-CoV-2 RNA and SARS-CoV-2 subgenomic RNA were assayed with quantitative RT-PCR as described in detail earlier (13).

Primers/probe sequences for SARS-CoV-2 RNA were as follows: 2019-nCoV_N1-F, 5′-GAC CCC AAA ATC AGC GAA AT-3′; 2019-nCoV_N1-R, 5′-TCT GGT TAC TGC CAG TTG AAT CTG-3′; 2019-nCoV_N1-P, 5′-FAM-ACC CCG CAT TAC GTT TGG TGG ACC-BHQ1-3′.

Primers/probe sequences for SARS-CoV-2 subgenomic RNA were as follows: sg-N-F, 5′-CGATCTCTTGTAGATCTGTTCTC-3′; sg-N-R, 5′-GGTGAACCAAGACGCAGTAT-3′; and Sg-N-P, 5′-6-FAM-TAACCAGAA-ZEN-TGGAGAACGCAGTGGG-3IABkFQ.

### (vi) Statistical analyses.

Overall, statistical analyses were conducted using a one-way ANOVA followed by Tukey's multiple-comparison tests. Detailed description of these analyses are provided in figure legends (Fig. 6 to 8).
